# Shifts of Antibiotic Resistomes in Soil Following Amendments of Antibiotics-Contained Dairy Manure

**DOI:** 10.3390/ijerph191710804

**Published:** 2022-08-30

**Authors:** Jijun Kang, Yiming Liu, Xiaojie Chen, Fei Xu, Wenguang Xiong, Xiubo Li

**Affiliations:** 1 Key Laboratory of Animal Antimicrobial Resistance Surveillance, Ministry of Agriculture and Rural Affairs, Feed Research Institute, Chinese Academy of Agricultural Sciences, Beijing 100081, China; 2 Guangdong Provincial Key Laboratory of Veterinary Pharmaceutic Development and Safety Evaluation, South China Agricultural University, Guangzhou 510642, China

**Keywords:** dairy manure, antibiotics, antibiotic resistance genes, microbial communities, soil

## Abstract

Dairy manure is a nutrition source for cropland soils and also simultaneously serves as a contamination source of antibiotic resistance genes (ARGs). In this study, five classes of antibiotics including aminoglycosides, beta-lactams, macrolides, sulfonamides, and tetracyclines, were spiked in dairy manure and incubated with soil for 60 days. The high throughput qPCR and 16S rRNA amplicon sequencing were used to detect temporal shifts of the soil antibiotic resistomes and bacterial community. Results indicated dairy manure application increased the ARG abundance by 0.5–3.7 times and subtype numbers by 2.7–3.7 times and changed the microbial community structure in soils. These effects were limited to the early incubation stage. Selection pressure was observed after the addition of sulfonamides. Bacterial communities played an important role in the shifts of ARG profiles and accounted for 44.9% of the resistome variation. The incubation period, but not the different antibiotic treatments, has a strong impact on the bacteria community. *Firmicutes* and *Bacteroidetes* were the dominant bacterial hosts for individual ARGs. This study advanced our understanding of the effect of dairy manure and antibiotics on the antibiotic resistome in soils and provided a reference for controlling ARG dissemination from dairy farms to the environment.

## 1. Introduction

Antibiotics exert an important role in protecting both human and animal health. In recent years, fast-growing antibiotic resistance has aroused global concern. Heavy use of antibiotics in animals was blamed as being a cause for the emergency and spread of antibiotic resistance genes (ARGs) [1]. It is predicted the global consumption of veterinary antibiotics will increase sustainably and achieve 104,079 tons in 2030 [2]. Moreover, around 40–95% of applied antibiotics were excreted into the environment by prototype or metabolites through feces and urine [3]. As the largest antibiotic consumer, China started the reduction action of antimicrobial agents used in veterinary practice in 2018, with the target of curbing animal-derived antibiotic resistance and its dissemination to the environment and humans [4].

The animal-derived antibiotic resistance not only comprises the therapeutic effect of antibiotics on animal disease but also poses a potential risk to human health. Tigecycline, the last antibiotic defense against serious infections in humans [5], has not been approved for animal use. However, the recent finding of *tet*X variants, such as *tet*(X4)*, tet*(X5)*, and tet*(X6), which could inactivate tigecycline and had been widely found in the bacteria of swine [6], poultry [7], and their surrounding environments [8]. This indicates that treatment for the infection of such ARG-hosted bacteria is likely to become extremely difficult for humans. Horizontal gene transfer, which is triggered by mobile genetic elements (MGEs), including plasmids, integrons, and transposons, could trigger and enhance the dissemination of ARGs among various bacterial populations and various species [9].

Land application of animal manure is a common measure to fertilize cropland soil. Simultaneously, it is considered a primary and direct path for veterinary antibiotic contaminants such as antibiotic residues, antibiotic-resistant bacteria, and ARGs, into the environment [10]. The manure of swine, poultry, and cattle had been reported with diverse ARGs [11]. ARGs in animal manure not only directly contaminate the receiving environments such as soil, groundwater, and rivers, but also indirectly contaminate cultivated plants, and could even be transmitted to humans through the food chain [12]. Understanding the ARG transmission mechanisms from animal manure to soil will be useful for improving the management practice of animal wastes and reducing the spread of animal-derived antibiotic resistance.

As one of the best protein sources, dairy cow production is expanding worldwide [13], correspondingly, leading to an increase in dairy manure production. Though the limitation of antibiotic usage is conducted in many countries, various antibiotics were used as a necessity in various growth stages of the dairy cow for disease prevention and treatment. Aminoglycosides, beta-lactams, macrolides, sulfonamides, and tetracyclines are the most universally adopted antibiotics both in humans and animals [14]. For example, beta-lactams are used to cure metritis in dairy cows and prophylactically eliminate intramammary infections during dry-off [15,16], and tetracyclines were applied as the first-line antibiotic to treat acute mastitis and respiratory disease in cows [17,18]. Antibiotics were assumed to exert selection pressure on the occurrence and dissemination of ARGs. Some surveys reported after clinical antibiotic administration to animals, that the collected manure can impact the ARGs profiles in soil [19,20]. However, the direct evidence of antibiotic selective pressure on ARGs and the bacteria community in dairy manure-amended soil is still less explored.

In the present study, we applied the dairy manure spike with or without five classes of common antibiotics into soils under the same lab conditions, we hypothesized that (i) dairy manure application could enhance the ARG abundance and diversity in farmland soil; (ii) the addition of different antibiotics could generate selection pressure, and the temporal dissipation of ARGs would be slower than un-treated groups; and (iii) the dynamics of ARGs can be driven by the changes in bacteria. We aim to understand the impact of dairy manure on the evolution of ARG profiles in receiving soil and the key factor for driving these changes, and provide a scientific basis for controlling the ARG dissemination from dairy manure to farmland.

## 2. Materials and Methods

### 2.1. Experimental Design and Sampling

The manure used in this study was obtained from a dairy farm located in north Beijing, China. The soil was collected from cropland located far from this farm. Both dairy manure and soil were air-dried at room temperature and sieved through 2 mm pores. A total of 21 plastic vessels were dispatched into seven groups in triplicate, including blank control soil without amendment (BS), soil + manure (BSM), soil + manure + aminoglycosides (gentamicin, kanamycin, neomycin, (AMI)), soil + manure + beta-lactams (amoxicillin, oxacillin, ceftiofur, (BETA)), soil + manure + macrolides (erythromycin, tylosin, tilmicosin, (MAC)), soil + manure + sulfonamides (sulfadimidine, sulfathiazole, sulfamethoxazole, (SUL)), and soil+ manure + tetracyclines (oxytetracycline, aureomycin, tetracycline, (TET)). 2000 g sieved soil with or without 80 g (*w*/*w*, 4%) manure was added to each pot [21]. The antibiotics were mixed evenly with dairy manure at the concentration of 5 mg/kg and then with soil [22]. All pots were kept dark at 25 °C. The water holding capacity of the soil was kept at 55% by adding tap water periodically [21]. The soil was sampled from each pot at three incubation stages (day 1, 30, and 60) and stored at −40 °C for further analysis.

### 2.2. DNA Extraction

The DNA of soil was extracted using Fast DNA^®^SPIN Kit for Soil (MPBIO, Santa Ana, CA, USA) following the manufacturer’s instructions [23]. The concentration of DNA was detected using Qubit 3.0 fluorescent ration PCR instrument (Thermo Fisher Scientific Inc., Waltham, MA, USA). The extracted DNA was kept at −20 °C for further analysis.

### 2.3. High-Throughput qPCR (HT-qPCR) for ARGs Analysis

A total of 80 PCR primer sets were selected to quantify the antibiotic resistomes, covering 15 aminoglycoside resistance genes, 15 beta-lactam resistance genes, 15 macrolide resistance genes, 4 sulfonamide resistance genes, 15 tetracycline resistance genes, 13 multidrug resistance genes, 2 integron genes and one 16S rRNA gene (Table S1). A 100 nL PCR reaction mixture containing 2 ng/μL DNA template, 500 nM of each primer, and 1 × LightCycler 480 SYBR Green IMaster, was conveyed to Wafergen Smartchip Real-time PCR system (MicroAnaly Genetech Co., Ltd., Anhui, China) for a run under the following procedure: 10 min at 95 °C for one cycle; 30 s at 95 °C, 30 s at 60 °C for 40 cycles. Three technical replicates and negative control were set up. The melting curve was used to check the specificity of the PCR outcome. The threshold of cycle value (CT) was 31. The range of amplification efficiency was 1.8–2.3. The relative gene copy number (N) is calculated by applying the following formula [24]:

N = 10^(31 − CT)/(10/3)^

The gene was considered to be detected only when the copy numbers of all three technical replicates were above the threshold. The relative abundance was expressed as the gene copy numbers per copy number of 16S rRNA.

### 2.4. High-Throughput Sequencing for Bacterial 16S rRNA

The V3-V4 region of the 16S rRNA gene was used for amplification with the primer 341F (5′-CCTACGGGNGGCWGCAG-3′) and 806R (5′-GACTACNVGGGTATCTAAT-3′) [25]. 25 μL PCR amplification mixture contained 10 ng DNA template, 10 μM of each primer, 12.5 μL of Phusion Hot start flex 2X Master Mix, and using 10 μg/μL BSA adjust to volume. PCR procedure was as follows: 94 °C for 3 min; 35 cycles of 94 °C for 30 s, 55 °C for 45 s, and 72 °C for 45 s; finally, 72 °C for 10 min. Purified PCR products were submitted for Illumina MiSeq PE300 platform (MicroAnaly Genetech Co., Ltd., Anhui, China). BBDuk (bbmap-37.75) [26] was used to trim amplicon sequencing data. FLASH 1.2.11 (Tanja Magoc, Baltimore, MD, USA) [27] was used to assemble the reads into raw tags. The high-quality clean tags were obtained and clustered using Vsearch 2.7.1 (Torbjørn Rognes, Oslo, Norway) [28]at the 97% similarity level. The generated operational taxonomic units (OTUs) were mapped against the SILVA database [29,30] at the 80% threshold to identify the bacteria. Before further analysis, downstream of OTUs was performed by QIIME 1.9.1 [31] using the standard of the smallest amount of data in the samples.

### 2.5. Statistical and Network Analysis

Statistical analysis was performed using SPSS software (version 26.2, SPSS Inc., Chicago, IL, USA). A significant difference was defined when *p* < 0.05. Principal component analysis (PCA) was performed based on the coverage of ARG types. Bacteria alpha diversity indexes, including observed OTUs, Chao1 index, Shannon index, and PD_Whole_tree index was calculated by QIIME1.9.1. Bacteria beta diversity (phylum level) was presented by principal coordinate analysis (PCoA) based on weighted-Unifrac matrix distance. Permutational multivariate analysis of variation (PERMANOVA, adonis) was used to compare the difference among groups. Spearman’s rank method was used to calculate the correlations between the ARGs and bacteria. PCA, shared genes comparison, PCoA, Procrustes analysis, redundancy analysis (RDA), mantel test, and variation partitioning analysis (VPA) were performed and visualized using R software 3.6.3 (R Core Team, Vienna, Austria). The network visualization was performed in Gephi (version 0.9.5, Mathieu Jacomy, Aalborg, Denmark). The heatmap of ARGs and other figures were created using ImageGP [32].

## 3. Results

### 3.1. Temporal Shifts of ARG Diversity and Abundance

**Figure 1:**
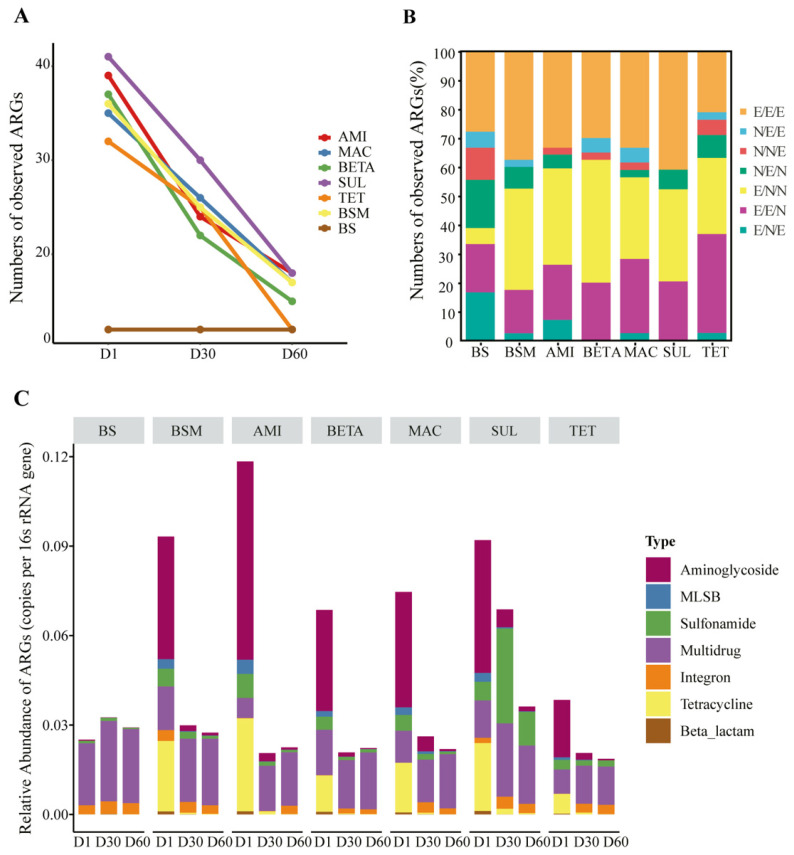
Temporal changes of ARG diversity and abundance. (**A**) The number of observed ARGs among different groups. (**B**) The succession of ARGs, expressed by the percentage of ARG numbers. E: exist; N: not exist; three letters orderly present the detect outcome of day 1, 30, and 60. (**C**) Relative abundance of ARGs in each sample (copies per 16s rRNA gene).

A total of 51 ARGs genes were detected among all the soil samples, ranging from 12–41 subtypes in different treatments (Figure 1A). These genes were predominantly resistant to aminoglycoside (29.41%), tetracycline (23.53%), macrolide-lincosamide-streptogramin B (MLSB) (15.69%) and multidrug (11.76%) (Figure S1). The BS harbored low and stable observed ARG numbers at three time points, while manure application significantly enhanced the ARG numbers by 2.7 times (TET) to 3.4 times (SUL) (*p* < 0.05) at the first incubation day and gradually decreased.

The relative abundance of detected individual ARGs ranged from 1.06 × 10^−5^ to 6.65 × 10^−2^ copies per 16S rRNA (Figure 1C). In BS, the total ARG abundance changed slightly during the whole incubation period, and was absolutely dominated by the genes resistant to multidrug, which accounted for 82.9–85.2% of total abundance. Compared with BS, great increases in total abundance were observed in all manure-contained groups by 0.5 times (TET) to 3.7 times at the early incubation stage (AMI) (*p* < 0.05). However, compared with the BSM, the addition of five classes of antibiotics did not change the total abundance in a significant way (*p* > 0.05). The manure-contained groups were predominant by the ARGs resistant to aminoglycoside (44.1–56.2%), tetracycline (17.5–26.3%), and multidrug (5.7–22.1%) on the first incubation day, and regained dominance of multidrug up to day 30 and 60, except for SUL group, of which the abundance of sulfonamide resistance genes was obviously higher on both day 30 and day 60 than day 1. It might be driven by the application of sulfonamide.

The succession of individual genes was tracked (Figure 1B). 21.05–40.91% of ARGs in different groups were persistent for 60 days. The following genes were persistent in not less than 5 groups, including *aad*A-01, *aad*A-02, *aad*A2-03, *str*B, *sul*2, *mex*F, *erm*F, and *tet*X. In BS, 22.2% of ARGs disappeared on day 30 whereas most reappeared on day 60, while 29.0–42.5% of ARGs disappeared in manure-contained groups and only a fraction reappeared. Up to day 60, more ARGs disappeared in the manure-contained groups (56.4–68.4%) than in the BS group (38.9%). However, there was no significant difference between BSM and antibiotic-contained groups for the disappeared ARG numbers (*p* < 0.05). The following genes were disappeared from not less than 5 groups, including *aph*A1(aka kanR), *aac*(6′)-Ib(aka aacA4)-03, *cfx*A, *mef*A, *lnu*B-01, *lnu*B-02, *dfr*A1, *tet*O-01, *tet*M-02, *tet*36-01, and *tet*H. Few new genes emerged on day 30 or day 60 in all groups and mainly belong to the class of multidrug and integron, such as *mep*A, *flo*R, *intl*-1(clinic), and *clntl*-1(class 1).

### 3.2. ARG Profiles and Detailed Composition

**Figure 2:**
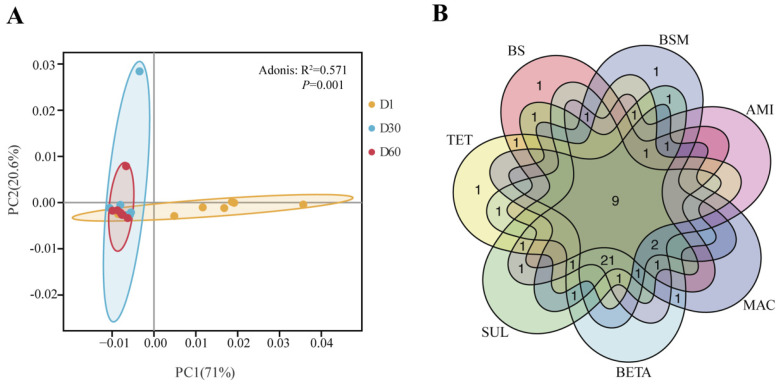
Comparison of ARG profiles among different samples. (**A**) PCA plots of different soil samples. (**B**) Shared ARGs across seven groups.

The similarity of ARG profiles of different samples was analyzed using PCA (Figure 2A). The manure-contained samples of day 1 presented scattered and independent distribution in the PC1 coordinate. It is revealed that manure application has a strong impact on ARG profiles of soils. All the rest of the samples of day 30 and day 60 cluster together with three BS samples, implying the similarity in their composition. The divergence of SUL samples of day 30 and day 60 was clearly shown in the PC2 coordinate. The shared and unique genes among the seven experimental groups were compared (Figure 2B). An amount of 9 genes were shared among all the groups, 21 genes were shared among manure-contained groups, and 7 genes were only found in antibiotic-contained groups while they did not display in both BS and BSM groups (Table S2). This outcome illustrated the ARG profile was more liable to be affected by manure application than antibiotic addition.

**Figure 3:**
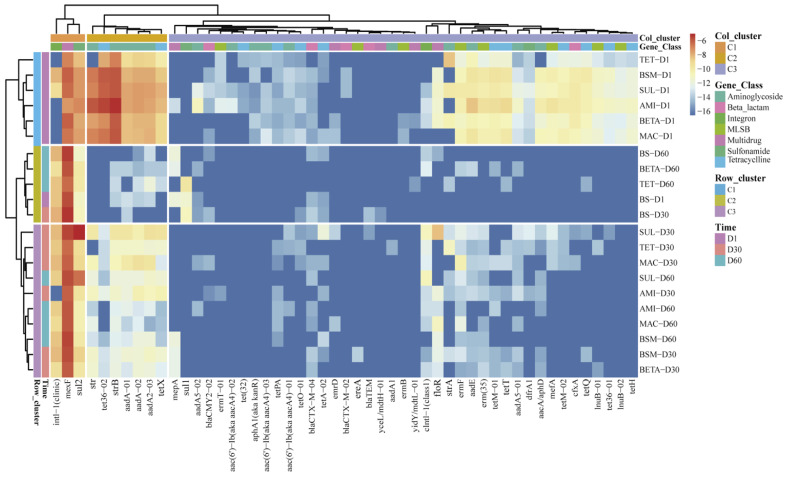
Heatmap of ARGs (coverage, copies per 16s rRNA gene, log^2^ transferred) of soil samples. Hierarchical clustering was based on Euclidean distance with the clustering method “complete”.

The heatmap shows the ARG profile of the individual sample, which was clustered according to the ARG distribution (Figure 3). From row clusters, we could see that cluster 1 covered all manure-contained samples of day 1, which possess the most ARG richness and abundance; BETA and TET of day 60 clustered together with three BS soils in cluster 2, showing their profiles were regressed and close to the background value of untreated soil; the third cluster could be considered to show that the effect of manure and antibiotic still continues. Individual genes in the column were also divided into three clusters: cluster 1 including *intl*-1(clinic), *mex*F, and *sul*2, these genes were the most abundant and almost existed in all samples; cluster 2 contained 7 genes that mainly convey resistance to aminoglycoside and tetracycline, the abundance of these genes increased after manure application and were persistent at a relatively high level throughout the experiment; cluster 3 contained 41 lower abundant or less detectable genes, most of their abundance decreased by a large margin or dissipated over time.

### 3.3. Dynamic Shifts of Bacterial Community

**Figure 4:**
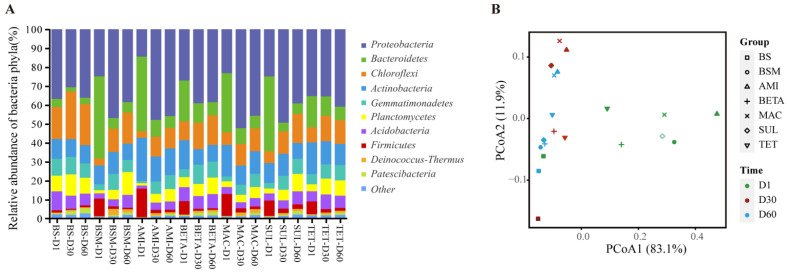
Bacterial abundance and diversity of different samples. (**A**) Relative abundance of major bacteria at the level of phylum. (**B**) PCoA plot of bacteria among different soil samples on day 1, 30, and 60.

The bacterial composition of different samples at the phylum level is shown in Figure 4A. *Proteobacteria*, *Bacteroidetes*, *Chlorofexi*, and *Actinobacteria* were the most preventable phyla among all samples. In BS, *Proteobacteria* was maintained as the dominant bacteria at all incubation stages and accounted for 30.7–36.8% of total relative abundance. Compared with BS, dairy manure application significantly increased the relative abundance of *Firmicutes* by 5.8 times (TET) to 12.9 times (AMI) and *Bacteroidetes* by 3.8 times (TET) to 10.0 times (BSM) (*p* < 0.05) on the first incubation day. These two bacteria, respectively, accounted for 16.5–43.4% and 6.7–15.1% of the phylum population, both of which decreased over time.

Through the comparison with observed OUT, Chao1 index, Shannon index, and PD_Whole_tree index (Figure S2) of BS, we found after dairy manure application, the bacteria alpha diversity was significantly decreased on the first incubation day (*p* < 0.05), and increased subsequently. However, compared with BSM, the antibiotic application did not change the alpha diversity substantially (*p* > 0.05). The bacteria beta diversity was analyzed using PCoA, the first two principal coordinates totally explained 95.0% of the bacterial variation (Figure 4B). Manure-contained samples were distributed independently from BS, and the samples of day 1 are separate from day 30 and day 60. PERMANOVA test showed incubation duration had a stronger impact on the bacterial structure (Adonis test, R^2^ = 0.56, *p* < 0.001) than different treatments (Adonis test, R^2^ = 0.23, *p* > 0.05).

### 3.4. Correlations between ARGs and Bacteria Communities

**Figure 5:**
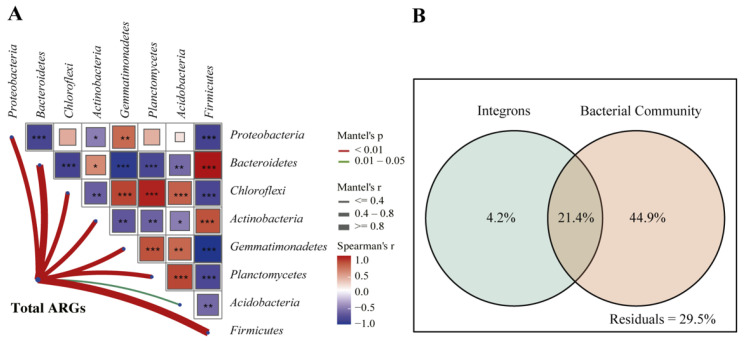
Drivers of the ARG profiles in the soil. (**A**) Mantel test between the entire ARG profiles and dominant bacterial community (>2% percentage). *, **, *** represent Spearman’s *p* < 0.05, *p* ≤ 0.01, *p* ≤ 0.001, respectively. (**B**) VPA chart showing the effects of bacterial community and integrons on the shifts of soil resistomes.

**Figure 6:**
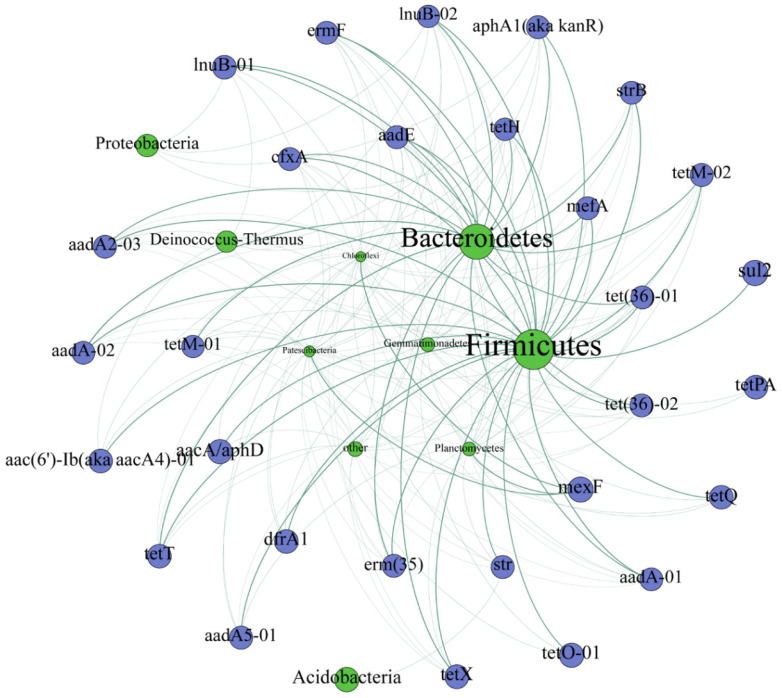
Network analysis between individual ARGs and bacteria community. The green and blue nodes represent the bacteria phyla and ARG subtype, respectively. A connection represents a strong correlation (Spearman’s correlation coefficient ρ ≥ 0.7 and significance level *p* < 0.01). The size of a node is proportional to the weighted degree, that is, the sum of the correlation coefficient of all connections related to this node.

Procrustes analysis indicated there was a significant correlation between the ARGs profiles and bacterial communities (M^2^ = 0.817, *p* = 0.001) (Figure S3). A Mantel test was performed between ARG profiles and the bacteria phyla that shared > 2% of the composition. The result revealed ARG profiles were most correlated with *Firmicutes* and *Bacteroidetes*, followed by *Gemmatimonadetes*, *Chloroflexi*, *Proteobacteria, Planctomycetes*, and *Actinobacteria* (*p* < 0.01) (Figure 5A). The correlation between ARG profiles (grouped by ARG type) and these seven bacteria phyla (mantel’s *p* < 0.01) was further performed using RDA. The outcome demonstrated genes resistant to aminoglycosides, beta-lactams, macrolides, and tetracyclines were positively correlated with *Firmicutes*, *Bacteroidetes*, and *Actinobacteria*, while genes resistant to multidrug were more correlated with *Gemmatimonadetes*, *Chloroflexi*, *Planctomycetes* and *Proteobacteria* (Figure S4). The outcome of VPA showed that 70.5% of ARG profile variance can be explained while bacteria communities contributed 44.9%, showing the bacterial community was the absolutely dominant factor affecting the ARG profiles (Figure 5B). The network analysis further investigated the co-occurrence of individual ARG and bacteria. A potential microbial host was defined if it was significantly associated with ARG (Spearman’s correlation ρ ≥ 0.7, *p* < 0.01). *Firmicutes* and *Bacteroidetes* were identified as the primary hosts of specific ARGs (Figure 6).

## 4. Discussion

### 4.1. Dairy Manure Application Enriched ARGs in Soil

In this study, an amount of 18 ARGs were detected in untreated soils (BS), but most had very low relative abundance. The multidrug resistance gene *mex*F and integron gene *intl*-1(class 1) dominated BS, and accounted for 81.7–84.1% and 12.2–12.9% of total relative abundance at three incubation stages, respectively. *Mex*F was detected in each sample with high abundance. It is considered to be the core ARG of soil and is more tolerant of external amendments [33]. As one of the most common MGEs, *intl*-1 could capture a variety of resistance genes and turn them into multidrug-resistant ones, further promoting the horizontal gene transfer of ARGs [34]. The universality of *mex*F and *intl*-1 was also found in groundwater [35]. It is revealed that these are two intrinsic genes in pristine environments. Notably, the sulfonamides resistant gene *sul*2 was detected in all BS samples and shared 1.3–1.9% of total relative abundance, which might be attributed to the genetic link between sulfonamides resistance genes and class1 integron. The co-occurrence of both kinds of genes was frequently reported [36,37].

Compared with BS, dairy manure application significantly increased the relative abundance and the numbers of ARGs in farmland soils, especially at the early incubation stage. Among the 51 detected ARGs in soils, 33 ARGs were found only in manure-contained groups while not in BS. The present results supported the previous outcome that dairy manure was a reservoir of ARGs [38], and ARGs could be mitigated from manure to soils [39]. Positioning of the abundance of dairy manure driven ARGs was aminoglycoside > tetracycline > sulfonamides > MLSB > beta_lactams. This may be related to the multifactor functioning of antibiotic usage history, primitive resistome of animal gut, and the physicochemical properties of antibiotic metabolites. For example, resistance genes to aminoglycoside were often found as the primary ARGs in cattle manure [40,41], and the instability of beta_lactams in manure may explain the less detection rate of beta_lactams resistance genes [16]. *Tet*X is an inactivation enzyme of tetracycline, its variants can confer resistance to human’s last antibiotic resort tigecycline, and have been widely spread on swine farms [6]. In this study, *tet*X was detected in all dairy manure-contained soil while not in BS, demonstrating the dissemination path of *tet*X from dairy manure to its receiving environments. These results hint at the need for manure management before land application.

### 4.2. Various Impacts of Antibiotic Addition on ARGs in Soil

Antibiotic usage on farms was considered an important factor in promoting the occurrence of ARGs [42]. In this study, five classes of antibiotics commonly used in the dairy farm were selected and spiked with dairy manure to investigate their potential influence on ARGs in the soil. The results showed that the addition of each class of antibiotic not only led to the changes of counterpart resistant genes but also other multiple ARGs. For example, aminoglycoside application increased the ARG relative abundance of aminoglycoside by 61.8%, simultaneously, MLSB, sulfonamide, and tetracycline resistance genes elevated by 48.5, 34.6, and 32.0%, and integrons and multidrug resistance genes decayed by 97.1 and 53.9%, respectively. A similar phenomenon was reported in the sediments from the bullfrog farm, the administration of aminoglycoside and beta-lactams caused multiple ARG pollution [43]. These suggest the complexity of ARG patterns in soil was not solely related to the different antibiotic treatments. For example, recombining ARGs in a plasmid can create a variety of ARG enrichment styles [43], and sometimes metals have more antibiotic resistance selection pressure than the employed antibiotic itself [44].

Compared with BSM, we found that different antibiotic-contained soil present upward or downward trends in the total ARG abundance at the early stage of incubation, whereas their ARGs compositions still remained similar and clustered closely with BSM (Figure 1C and Figure 3). It is assumed that the addition of antibiotics only impacts certain carriers for entire ARG profiles, but independently from the specific ARG composition. Previous surveys also reported the tetracycline resistance genes in cattle manure amended soil were not significantly affected by the spike of chlortetracycline [45]. The level of ARGs in most manure-contained soils descended dramatically before day 30 and slightly changed before day 60 (Figure 1C). The ARG profiles of BETA and TET almost reverted to the BS on day 60, while slight differences still existed in other groups (Figure 3). These indicated the intensive impact of manure and antibiotic on these four groups was limited to a short time right after the addition. Zhang et al. [41] and Macedo et al. [46] reported similar findings, despite the observed periods (20 to 130 days) back to the untreated level being different. This phenomenon might be credited to the quick transfer of ARGs carrier from manure to soil at the start, whereas the resilience of soil varies under different treatments.

Selective pressure was only observed in the SUL group, of which sulfonamides resistant genes were obviously more abundant on day 30 and day 60 (5.1 and 1.8 times) than on day 1. Sulfonamides have high mobility and are potentially liable to transfer from soil to groundwater [47]. Previous literature revealed that manure application can enhance the sorption of sulfonamides in soil and delay its mobility [48]. After being used in the soil for 28 days, Sulfamethoxazole and its metabolites are still present in the soil [49]. In addition, some bacteria could provide a suitable microenvironment for sulfonamides survival and are less degraded [50]. The potentially persistent existence of sulfonamides in the soil might explain the boosting occurrence of sulfonamides resistant genes in our study. These results support the suggestion of restriction for the application of sulfonamide-contaminated dairy manure into farmland soil, to reduce the spread risk of ARGs.

### 4.3. Shifts of Bacterial Communities were the Primary Driver for the Changes of ARGs in Soil

In the present study, the BS microbial phyla were dominated by *Proteobacteria* and *Chloroflexi*. Both of them were often found as the main phyla in soils [51,52]. After the dairy manure application, the bacterial community structure in the soil was significantly changed by increasing the relative abundance of *Firmicutes* and *Bacteroidetes*, consistent with previous findings [53]. *Firmicutes* and *Bacteroidetes* had been widely reported as the predominant phyla in cattle manure, both accounted for approximately 70% of the bacterial community in some cattle fecal samples [3,54]. In agreement with previous findings [24], we found such shifts are rapid. The humid environment might facilitate the spread of microorganisms from manure to the soil [53], and the nutrients in manure such as the content of nitrogen and organic carbon could promote the proliferation of certain bacteria in soil [51]. The incubation period, not the addition of antibiotics, was the primary factor for the temporal shifts of the bacteria community in soil. Liu et al. [55] also reported the diversity and the abundance of the bacterial community were directly impacted by duration time, while without significant correlation with the different manure and treatments. Time restriction before manure application to cropland soil might be a simple and effective way to curb the dissemination of ARGs from dairy farms.

The shifts in the bacterial community, especially the ARG-carrying bacterial hosts, play a dominant role in shaping the ARG profiles [56,57], and our result echoed this opinion (Figure 5 and Figure 6). The primary ARG bacterial hosts in dairy manure-contained soils are *Firmicutes* and *Bacteroidetes* (Figure 6), which were widely documented as the hosts of multiple ARGs in different matrices such as feces of swine [58], poultry [59], and humans [25]. We found the change of composition percentage of these two phyla in soil was accompanied by the change in the total abundance of ARGs (Figure S5). For example, TET of day 1 had the lowest amount of *Bacteroidetes* and *Firmicutes* and the lowest amount of ARGs among all manure-contained groups. Composting could decrease the abundance of *Firmicutes* but increase the abundance of *Bacteroidetes* [59,60]. Measures to remove multiple ARG bacterial hosts should be further explored. MGEs were frequently identified as another driver for shaping ARG profiles [39,61]. However, they only explained a small portion of the ARG variation in this study. This might be due to only two integrons being investigated. More plasmids, transposons, and integrons genes should be surveyed in the future.

## 5. Conclusions

In summary, dairy manure can enrich the soil resistomes and change the bacterial community structure. Its intensive effect on the ARG profile occurred right after the application (on day 1) and persisted for a short time (ARG abundance descended dramatically before day 30). Time restriction of dairy manure application might be a simple and effective method to reduce the ARG dissemination risk. Sulfonamides can cause selection pressure for ARG in soils and last over 60 days. Hence, sulfonamide-contaminated manure should not be allowed on farmland. Shifts of antibiotic resistome were mainly driven by changes in the bacterial community, which was predominantly impacted by the manure application and incubation period, but not the addition of antibiotics. *Firmicutes* and *Bacteroidetes* are the most important ARG hosts of dairy manure. Further surveillance and research on attenuation technology of ARG bacterial hosts would be helpful to control the spread of ARGs from dairy manure into cropland soil.

## Data Availability

The 16s RNA sequence data that support the findings of this study have been deposited in the NCBI Sequence Read Archive (Accession No. PRJNA865385).

## Supplementary Materials

The following supporting information can be downloaded at: https://www.mdpi.com/article/10.3390/ijerph191710804/s1, Figure S1: The percentage of detected different ARG types; Figure S2: The alpha diversities of bacteria under seven treatments; Figure S3: Procrustes analysis between ARG profiles and bacterial community; Figure S4: RDA analysis between ARG types and dominant bacteria; Figure S5: Dynamic shifts of relative abundance of *Firmicutes*, *Bacteroidetes*, and total ARGs; Table S1: The information of ARGs for HT-qPCR. Table S2: Shared and exclusive genes among seven treatments.
