# Supplementary material for: Shifts of Antibiotic Resistomes in Soil Following Amendments of Antibiotics-Contained Dairy Manure
Source: Int J Environ Res Public Health. 2022 Aug 30;19(17):10804. doi: 10.3390/ijerph191710804 (PMC9517759; doi:10.3390/ijerph191710804)
Supplement: Supplementary file 1 [file ijerph-19-10804-s001.zip › ijerph-1852146-supplementary.pdf]

Supplementary data

Shifts of Antibiotic Resistomes in Soil Following Amendments of Antibiotics-Contained Dairy Manure

Jijun Kang [1,†], Yiming Liu [1,†], Xiaojie Chen [1], Fei Xu [1], Wenguang Xiong [2,*] and Xiubo Li [1,*]

1 Key Laboratory of Animal Antimicrobial Resistance Surveillance, Ministry of Agriculture and Rural Affairs, Feed Research Institute, Chinese Academy of Agricultural Sciences, Beijing 100081, China
2 Guangdong Provincial Key Laboratory of Veterinary Pharmaceutic Development and Safety Evaluation, South China Agricultural University, Guangzhou 510642, China
* Correspondence: xiongwg@scau.edu.cn (W.X.); lixiubo@caas.cn (X.L.)
† These authors have contributed equally to this work.

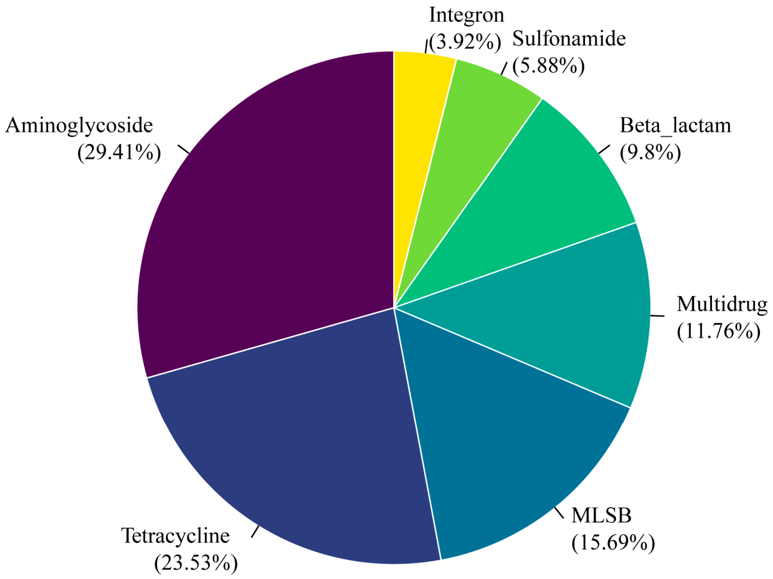

Figure S1. The percentage of detected different ARG types. MLSB: macrolide-lincosamide-Streptogramin B.

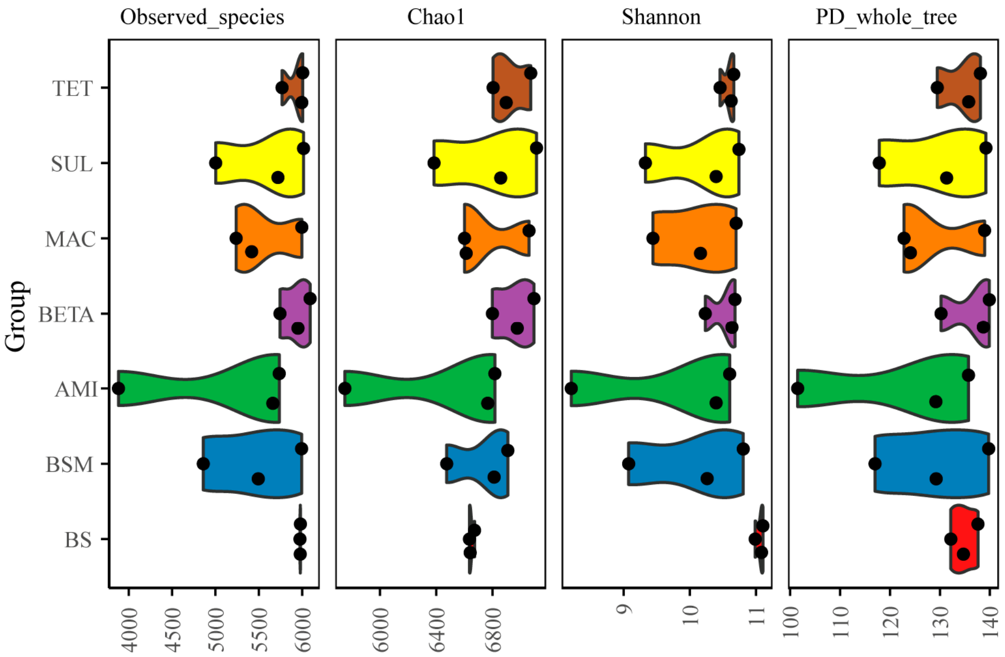

Figure S2. The alpha diversities of bacteria under seven treatments. The three nodes from left to right in each violin represent the Shannon index of day 1, 30, and 60. BS: blank control soil; BSM: soil+ dairy manure; AMI, BETA, MAC, SUL, and TET represent the soil with amended dairy manure by addition of aminoglycosides, beta-lactams, macrolides, sulfonamides and tetracyclines respectively.

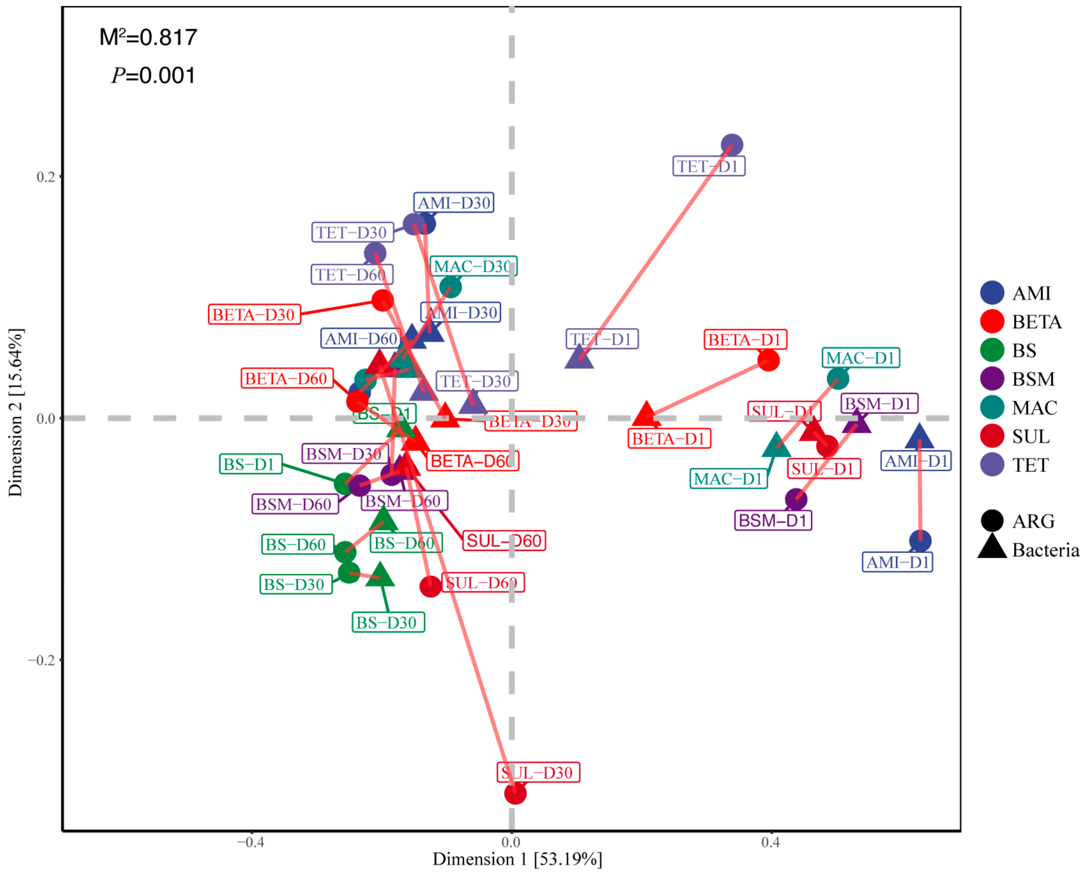

Figure S3. Procrustes analysis between ARG profiles and bacterial community.

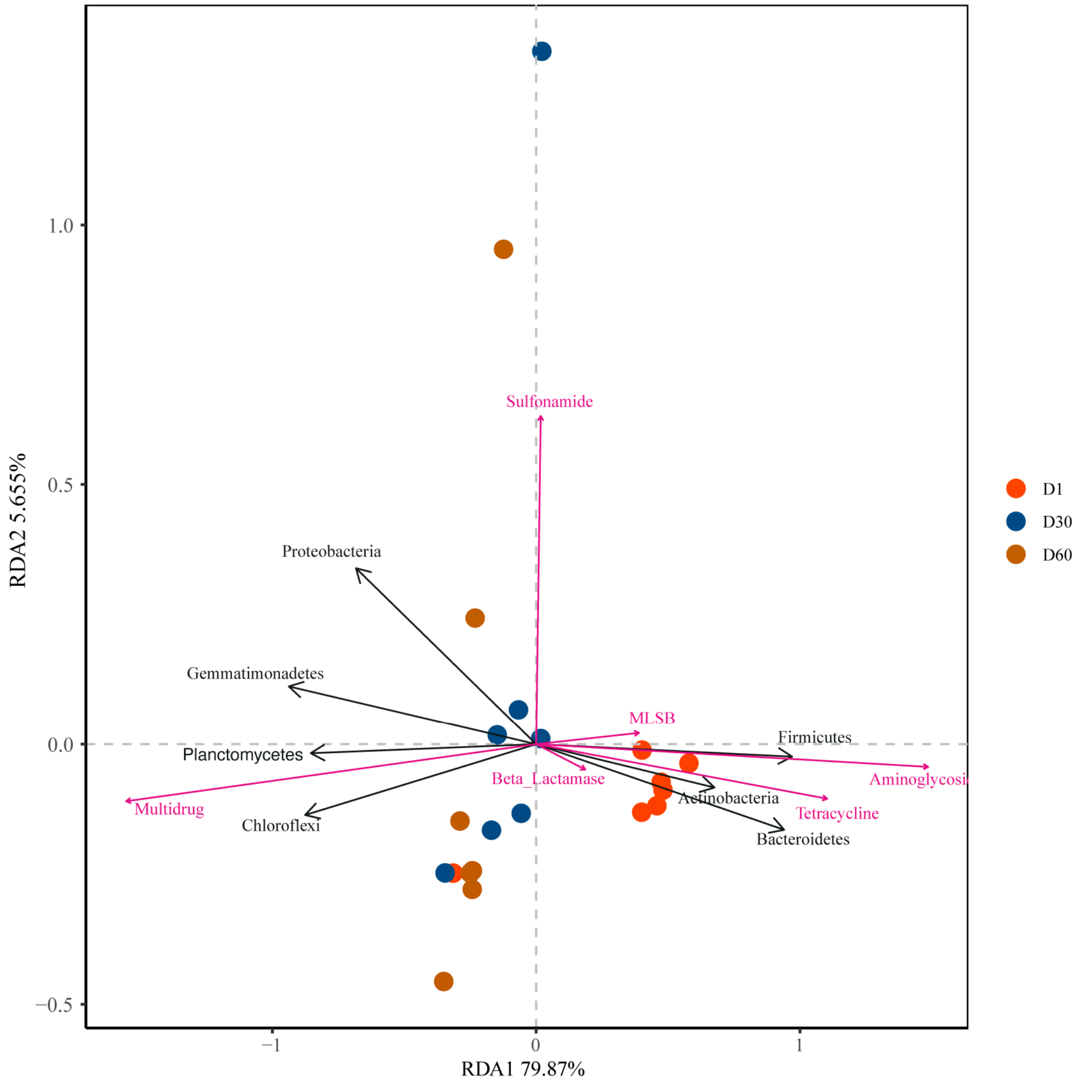

Figure S4. RDA analysis between ARG types and dominant bacteria.

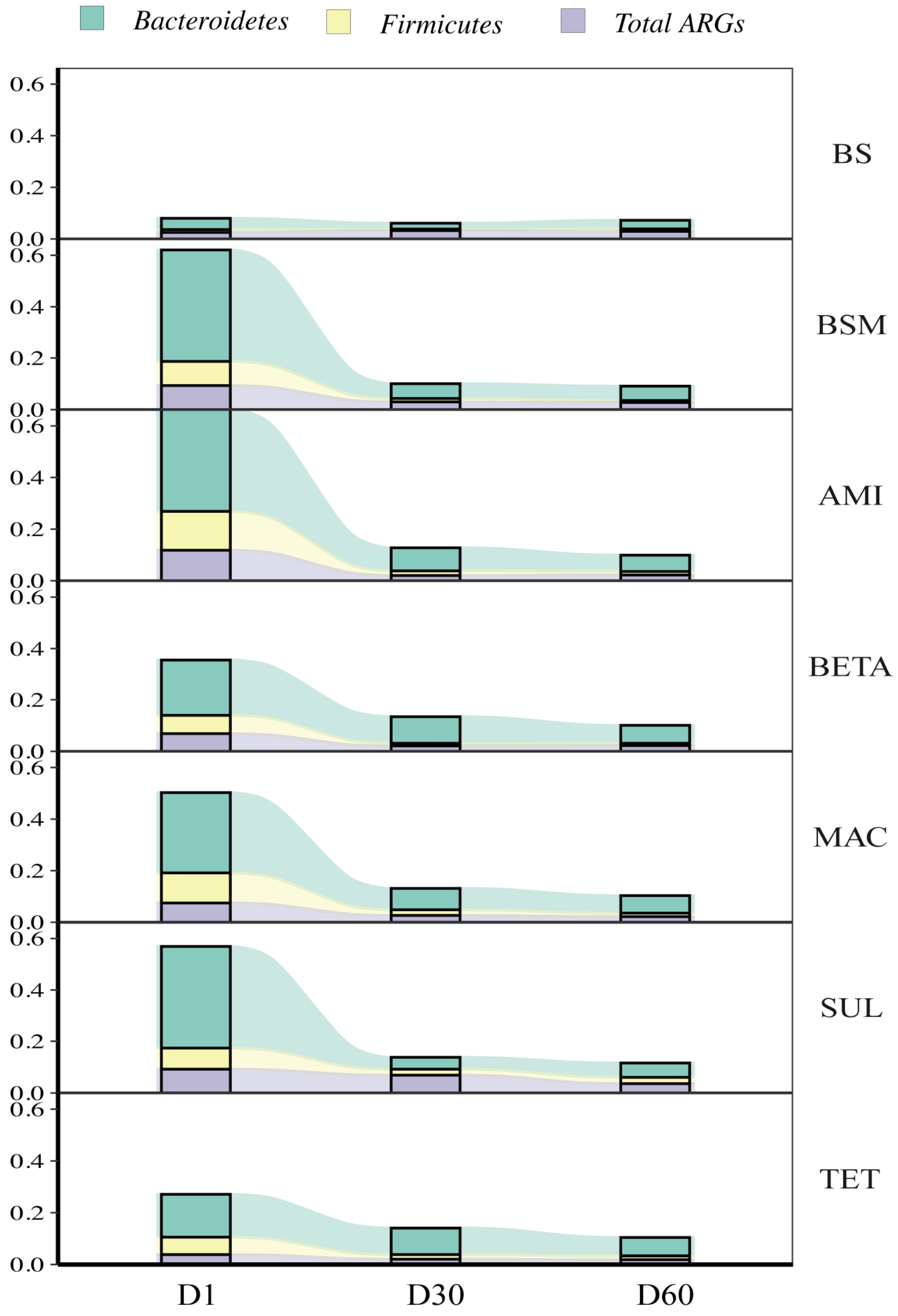

Figure S5. Dynamic shifts of relative abundance of *Firmicutes*, *Bacteroidetes,* and total ARGs.

Table S1. The information of antibiotic resistance genes (ARGs) for HT-qPCR.

No.	Assay Name	Forward Primer (5' to 3')	Reverse Primer (5' to 3')	Classification	Mechanism
1	16S rRNA gene	GGGTTGCGCTCGTTGC	ATGGYTGTCGTCAGCTCGTG		
2	*aac*(6')-Ib(aka aacA4)-01	GTTTGAGAGGCAAGGTACCGTAA	GAATGCCTGGCGTGTTTGA	Aminoglycoside	Deactivate
3	*aac*(6')-Ib(aka aacA4)-02	CGTCGCCGAGCAACTTG	CGGTACCTTGCCTCTCAAACC	Aminoglycoside	Deactivate
4	*aac*(6')-Ib(aka aacA4)-03	AGAAGCACGCCCGACACTT	GCTCTCCATTCAGCATTGCA	Aminoglycoside	Deactivate
5	*aacA/aph*D	AGAGCCTTGGGAAGATGAAGTTT	TTGATCCATACCATAGACTATCTCATCA	Aminoglycoside	Deactivate
6	*aad*A-01	GTTGTGCACGACGACATCATT	GGCTCGAAGATACCTGCAAGAA	Aminoglycoside	Deactivate
7	*aad*A-02	CGAGATTCTCCGCGCTGTA	GCTGCCATTCTCCAAATTGC	Aminoglycoside	Deactivate
8	*aad*A1	AGCTAAGCGCGAACTGCAAT	TGGCTCGAAGATACCTGCAA	Aminoglycoside	Deactivate
9	*aad*A2-03	CAATGACATTCTTGCGGGTATC	GACCTACCAAGGCAACGCTATG	Aminoglycoside	Deactivate
10	*aad*A5-01	ATCACGATCTTGCGATTTTGCT	CTGCGGATGGGCCTAGAAG	Aminoglycoside	Deactivate
11	*aad*A5-02	GTTCTTGCTCTTGCTCGCATT	GATGCTCGGCAGGCAAAC	Aminoglycoside	Deactivate
12	*aad*E	TACCTTATTGCCCTTGGAAGAGTTA	GGAACTATGTCCCTTTTAATTCTACAATCT	Aminoglycoside	Deactivate
13	*aph*A1(aka kanR)	TGAACAAGTCTGGAAAGAAATGCA	CCTATTAATTTCCCCTCGTCAAAAA	Aminoglycoside	Deactivate
14	*str*	AATGAGTTTTGGAGTGTCTCAACGTA	AATCAAAACCCCTATTAAAGCCAAT	Aminoglycoside	Deactivate
15	*str*A	CCGGTGGCATTTGAGAAAAA	GTGGCTCAACCTGCGAAAAG	Aminoglycoside	Deactivate
16	*str*B	GCTCGGTCGTGAGAACAATCT	CAATTTCGGTCGCCTGGTAGT	Aminoglycoside	Deactivate
17	*bla*CMY	CCGCGGCGAAATTAAGC	GCCACTGTTTGCCTGTCAGTT	Beta_Lactamase	Deactivate
18	*bla*CMY2-01	AAAGCCTCAT GGGTGCATAAA	ATAGCTTTTGTTTGCCAGCATCA	Beta_Lactamase	Deactivate
19	*bla*CMY2-02	GCGAGCAGCCTGAAGCA	CGGATGGGCTTGTCCTCTT	Beta_Lactamase	Deactivate
20	*bla*CTX-M-01	GGAGGCGTGACGGCTTTT	TTCAGTGCGATCCAGACGAA	Beta_Lactamase	Deactivate

21	*bla*CTX-M-02	GCCGCGGTGCTGAAGA	ATCGGATTATAGTTAACCAGGTCAGATTT	Beta_Lactamase	Deactivate
22	*bla*CTX-M-03	CGATACCACCACGCCGTTA	GCATTGCCCAACGTCAGATT	Beta_Lactamase	Deactivate
23	*bla*CTX-M-04	CTTGGCGTTGCGCTGAT	CGTTCATCGGCACGGTAGA	Beta_Lactamase	Deactivate
24	*bla*CTX-M-05	GCGATAACGTGGCGATGAAT	GTCGAGACGGAACGTTTCGT	Beta_Lactamase	Deactivate
25	*bla*CTX-M-06	CACAGTTGGTGACGTGGCTTAA	CTCCGCTGCCGGTTTTATC	Beta_Lactamase	Deactivate
26	*bla*SHV-01	TCCCATGATGAGCACCTTTAAA	TTCGTCACCGGCATCCA	Beta_Lactamase	Deactivate
27	*bla*SHV-02	CTTTCCCATGATGAGCACCTTT	TCCTGCTGGCGATAGTGGAT	Beta_Lactamase	Deactivate
28	*bla*TEM	AGCATCTTACGGATGGCATGA	TCCTCCGATCGTTGTCAGAAGT	Beta_Lactamase	Deactivate
29	*bla*Z	GGAGATAAAGTAACAAATCCAGTTAGATATGA	TGCTTAATTTTCCATTTGCGATAAG	Beta_Lactamase	Deactivate
30	*cfx*A	TCATTCCTCGTTCAAGTTTTCAGA	TGCAGCACCAAGAGGAGATGT	Beta_Lactamase	Deactivate
31	*mec*A	GGTTACGGACAAGGTGAAATACTGAT	TGTCTTTTAATAAGTGAGGTGCGTTAATA	Beta_Lactamase	Protection
32	*car*B	GGAGTGAGGCTGACCGTAGAAG	ATCGGCGAAACGCACAAA	MLSB	Efflux
33	*ere*A	CCTGTGGTACGGAGAATTCATGT	ACCGCATTCGCTTTGCTT	MLSB	Deactivate
34	*erm*(35)	TTGAAAACGATGTTGCATTAAGTCA	TCTATAATCACAACTAACCACTTGAACGT	MLSB	Protection
35	*erm*B	TAAAGGGCATTTAACGACGAAACT	TTTATACCTCTGTTTGTTAGGGAATTGAA	MLSB	Protection
36	*erm*C	TTTGAAATCGGCTCAGGAAAA	ATGGTCTATTTCAATGGCAGTTACG	MLSB	Protection
37	*erm*F	CAGCTTTGGTTGAACATTTACGAA	AAATTCCTAAAATCACAACCGACAA	MLSB	Protection
38	*erm*T-01	GTTCACTAGCACTATTTTTAATGACAGAAGT	GAAGGGTGTCTTTTTAATACAATTAACGA	MLSB	Protection
39	*erm*T-02	GTAAAATCCCTAGAGAATACTTTCATCCA	TGAGTGATATTTTTGAAGGGTGTCTT	MLSB	Protection
40	*erm*Y	TTGTCTTTGAAAGTGAAGCAACAGT	TAACGCTAGAGAACGATTTGTATTGAG	MLSB	Protection
41	*lnu*B-01	TGAACATAATCCCCTCGTTTAAAGAT	TAATTGCCCTGTTTCATCGTAAATAA	MLSB	Deactivate
42	*lnu*B-02	AAAGGAGAAGGTGACCAATACTCTGA	GGAGCTACGTCAAACAACCAGTT	MLSB	Deactivate

43	*lnu*C	TGGTCAATATAACAGATGTAAACCAGATTT	CACCCCAGCCACCATCAA	MLSB	Deactivate
44	*mdt*A	CCTAACGGGCGTGACTTCA	TTCACCTGTTTCAAGGGTCAAA	MLSB	Efflux
45	*mef*A	CCGTAGCATTGGAACAGCTTTT	AAACGGAGTATAAGAGTGCTGCAA	MLSB	Efflux
46	*mph*B	CGCAGCGCTTGATCTTGTAG	TTACTGCATCCATACGCTGCTT	MLSB	Deactivate
47	*acr*B-01	AGTCGGTGTTCGCCGTTAAC	CAAGGAAACGAACGCAATACC	Multidrug	Efflux
48	*acr*F	GCGGCCAGGCACAAAA	TACGCTCTTCCCACGGTTTC	Multidrug	Efflux
49	*emr*D	CTCAGCAGTATGGTGGTAAGCATT	ACCAGGCGCCGAAGAAC	Multidrug	
50	*flo*R	ATTGTCTTCACGGTGTCCGTTA	CCGCGATGTCGTCGAACT	Multidrug	Efflux
51	*mep*A	ATCGGTCGCTCTTCGTTCAC	ATAAATAGGATCGAGCTGCTGGAT	Multidrug	Efflux
52	*mex*F	CCGCGAGAAGGCCAAGA	TTGAGTTCGGCGGTGATGA	Multidrug	Efflux
53	*yce*E/*mdt*G-01	TGGCACAAAATATCTGGCAGTT	TTGTGTGGCGATAAGAGCATTAG	Multidrug	Efflux
54	*yce*E/*mdt*G-02	TTATCTGTTTTCTGCTCACCTTCTTTT	GCGTGGTGACAAACAGGCTTA	Multidrug	Efflux
55	*yce*L/*mdt*H-01	TCGGGATGGTGGGCAAT	CGATAACCGAGCCGATGTAGA	Multidrug	Efflux
56	*yce*L/*mdt*H-02	CGCGTGAAACCTTAAGTGCTT	AGACGGCTAAACCCCATATAGCT	Multidrug	Efflux
57	*yce*L/*mdt*H-03	CTGCCGTTAAATGGATGTATGC	ACTCCAGCGGGCGATAGG	Multidrug	Efflux
58	*yid*Y/*mdt*L-01	GCAGTTGCATATCGCCTTCTC	CTTCCCGGCAAACAGCAT	Multidrug	Efflux
59	*yid*Y/*mdt*L-02	TGCTGATCGGGATTCTGATTG	CAGGCGCGACGAACATAAT	Multidrug	Efflux
60	*dfr*A1	GGAATGGCCCTGATATTCCA	AGTCTTGCGTCCAACCAACAG	Sulfonamide	Deactivate
61	*dfr*A12	CCTCTACCGAACCGTCACACA	GCGACAGCGTTGAAACAACTAC	Sulfonamide	Deactivate
62	*sul*1	CAGCGCTATGCGCTCAAG	ATCCCGCTGCGCTGAGT	Sulfonamide	Protection
63	*sul*2	TCATCTGCCAAACTCGTCGTTA	GTCAAAGAACGCCGCAATGT	Sulfonamide	Protection
64	*tet*32	CCATTACTTCGGACAACGGTAGA	CAATCTCTGTGAGGGCATTTAACA	Tetracycline	Protection

65	*tet*36-01	AGAATACTCAGCAGAGGTCAGTTCCT	TGGTAGGTCGATAACCCGAAAAT	Tetracycline	Protection
66	*tet*36-02	TGCAGGAAAGACCTCCATTACAG	CTTTGTCCACACTTCCACGTACTATG	Tetracycline	Protection
67	*tet*A-01	GCTGTTTGTTCTGCCGGAAA	GGTTAAGTTCCTTGAACGCAAACT	Tetracycline	Efflux
68	*tet*A-02	CTCACCAGCCTGACCTCGAT	CACGTTGTTATAGAAGCCGCATAG	Tetracycline	Efflux
69	*tet*C-01	CATATCGCAATACATGCGAAAAA	AAAGCCGCGGTAAATAGCAA	Tetracycline	Efflux
70	*tet*C-02	ACTGGTAAGGTAAACGCCATTGTC	ATGCATAAACCAGCCATTGAGTAAG	Tetracycline	Efflux
71	*tet*H	TTTGGGTCATCTTACCAGCATTAA	TTGCGCATTATCATCGACAGA	Tetracycline	Efflux
72	*tet*M-01	CATCATAGACACGCCAGGACATAT	CGCCATCTTTTGCAGAAATCA	Tetracycline	Protection
73	*tet*M-02	TAATATTGGAGTTTTAGCTCATGTTGATG	CCTCTCTGACGTTCTAAAAGCGTATTAT	Tetracycline	Protection
74	*tet*O-01	ATGTGGATACTACAACGCATGAGATT	TGCCTCCACATGATATTTTTCCT	Tetracycline	Protection
75	*tet*PA	AGTTGCAGATGTGTATAGTCGTAAACTATCTATT	TGCTACAAGTACGAAAACAAAACTAGAA	Tetracycline	Efflux
76	*tet*Q	CGCCTCAGAAGTAAGTTCATACACTAAG	TCGTTCATGCGGATATTATCAGAAT	Tetracycline	Protection
77	*tet*T	CCATATAGAGGTTCCACCAAATCC	TGACCCTATTGGTAGTGGTTCTATTG	Tetracycline	Protection
78	*tet*X	AAATTTGTTACCGACACGGAAGTT	CATAGCTGAAAAAATCCAGGACAGTT	Tetracycline	Other/unknown
79	*clntl*-1(class1)	GGCATCCAAGCAGCAAG	AAGCAGACTTGACCTGA	Integron	integrase
80	*intl*-1(clinic)	CGAACGAGTGGCGGAGGGTG	TACCCGAGAGCTTGGCACCCA	Integron	Integrase

Table S2. Shared and exclusive genes among seven treatment.

Modules	ARG subtypes	ARG types
9 genes shared among all experimental groups	*aad*A-02, *aad*A2-03, *aad*A-01, *str*B, *intl*-1(clinic), *clntl*-1(class1), *mex*F, *sul*2, *tet*O-01	Aminoglycoside, multidrug, sulfonamide, tetracycline, Integron
21 genes shared among all manure-contained groups	*aad*A5-0*1*, *aad*E, *aac*A, *aph*D, *aac*(6')-Ib(aka aacA4)-01, *aph*A1(aka kanR), *cfx*A, *erm*F, *erm*35, *mef*A, *lnu*B-01, *lnu*B-02, *dfr*A1, *tet*X, *tet*36-02, *tet*M-01, *tet*PA, *tet*T, *tet*M-02, *tet*Q, *tet*36-01, *tet*H	Aminoglycoside, beta_lactam, multidrug, MLSB sulfonamide, tetracycline, Integron
7 genes exclusive for antibiotic-contained groups	*aac*(6')-Ib, (aka aacA4)-03, *tet*32, *yid*Y/*mdt*L-01, *aad*A1, *emr*D, *aac*(6')-Ib(aka aacA4)-02, *erm*B	Aminoglycoside, tetracycline, multidrug, MLSB
